# Angiogenin in the Neurogenic Subventricular Zone After Stroke

**DOI:** 10.3389/fneur.2021.662235

**Published:** 2021-06-21

**Authors:** Marina Gabriel-Salazar, Ting Lei, Alba Grayston, Carme Costa, Esperanza Medina-Gutiérrez, Manuel Comabella, Joan Montaner, Anna Rosell

**Affiliations:** ^1^Neurovascular Research Laboratory and Neurology Service, Vall d'Hebron Research Institute, Universitat Autònoma de Barcelona, Barcelona, Spain; ^2^Servei de Neurologia-Neuroimmunologia, Centre d'Esclerosi Múltiple de Catalunya (Cemcat) and Vall d'Hebron Research Institute, Hospital Vall d'Hebron, Universitat Autònoma de Barcelona, Barcelona, Spain

**Keywords:** stroke, angiogenin, neural stem/progenitor cells, neurogenesis, neurorepair, exercise

## Abstract

Ischemic stroke is a leading cause of death and disability worldwide with effective acute thrombolytic treatments. However, brain repair mechanisms related to spontaneous or rehabilitation-induced recovery are still under investigation, and little is known about the molecules involved. The present study examines the potential role of angiogenin (ANG), a known regulator of cell function and metabolism linked to neurological disorders, focusing in the neurogenic subventricular zone (SVZ). Angiogenin expression was examined in the mouse SVZ and in SVZ-derived neural stem cells (NSCs), which were exposed to exogenous ANG treatment during neurosphere formation as well as in other neuron-like cells (SH-SY5Y). Additionally, male C57Bl/6 mice underwent a distal permanent occlusion of the middle cerebral artery to study endogenous and exercise-induced expression of SVZ-ANG and neuroblast migration. Our results show that SVZ areas are rich in ANG, primarily expressed in DCX+ neuroblasts but not in nestin+NSCs. *In vitro*, treatment with ANG increased the number of SVZ-derived NSCs forming neurospheres but could not modify SH-SY5Y neurite differentiation. Finally, physical exercise rapidly increased the amount of endogenous ANG in the ipsilateral SVZ niche after ischemia, where DCX-migrating cells increased as part of the post-stroke neurogenesis process. Our findings position for the first time ANG in the SVZ during post-stroke recovery, which could be linked to neurogenesis.

## Introduction

Stroke affects 15 million people worldwide annually, and it is a leading cause of long-term disability in industrialized countries (1, 2). Thrombolytic and endovascular thrombectomy are the only available treatments during the acute phase of ischemic stroke to reduce mortality and minimize functional and motor disabilities (3–5). However, the narrow time window limits these strategies, and only a small number of patients benefit from them. Although these vessel-recanalization strategies are effective, a large percentage of stroke survivors still suffer from motor disabilities and neurological deficits. With this scenario, the only proven effective treatment for disabled stroke patients is rehabilitation, which aims to compensate for the affected sensory-motor function and improve life quality and independence for daily activities (6, 7). In spite of the proven benefits of multidisciplinary rehabilitation programs, these do not guarantee complete recovery for all patients, and individuals exhibit variable responses to similar treatments (8). In this regard, the biological responses responsible for the individual functional improvements have been investigated to identify brain plasticity mechanisms and targets to modulate the natural evolution of brain repair by rehabilitation (9–11), but are not fully elucidated. In this regard, a few pre-clinical studies have associated rehabilitation with restorative brain plasticity, including mechanisms of neuroangiogenesis (12–16); however, current knowledge on the molecules modulated by rehabilitation and potentially associated with brain plasticity is incomplete.

To further explore the molecular implications of tissue repair, we have focused on angiogenin (ANG), a ribonuclease protein that promotes cell proliferation and migration (17, 18) and related to excitotoxic motoneuron death in angiogenin loss-of-function mutations associated with ALS (19). We have previously shown ANG role in secretome-based therapies on brain endothelial cells (20), demonstrated the ANG upregulation in the blood of stroke patients under rehabilitation related to better outcomes at long term, and the angiogenin mRNA overexpression in the infarct tissue of ischemic mice also after rehabilitation (21). However, previous studies reported that neamine treatment (a blocking agent of the ANG activity) was neuroprotective after stroke in a rat model of type I diabetic rats and that the failure of bone-marrow-derived cell therapy after stroke in the same model of diabetic rats was potentially linked to an increase in periinfarct and vascular ANG in infiltrating macrophages (22, 23). Non-diabetic animals did not show ANG expression linked to vascular dysfunction (23). Its implications in neurogenesis are still unknown. In the present study, we aimed at investigating ANG in the subventricular zone (SVZ) niche after stroke by studying its tissue expression, effects on SVZ-derived neural stem cells (NSC), and its modulation after cerebral ischemia and rehabilitation.

## Materials and Methods

### Brain Tissue Samples

All animals used in the present investigation are C57BL/6 male mice (6–12 weeks old). To investigate SVZ neurogenesis and ANG cellular expression, we used brain tissue slices from a previous protocol in which cerebral ischemia was induced also by the permanent electrocauterization of the distal branch of the middle cerebral artery and mice were submitted to physical exercise rehabilitation (*n* = 6) or non-rehabilitation (*n* = 6) (21). New mice were also used to obtain SVZ tissues in naive animals (*n* = 4) or after ischemia/rehabilitation (treadmill, *n* = 6) or ischemia/non-rehabilitation (*n* = 6). Finally, frozen NSC obtained from mouse SVZ cultures following published protocols (24) were also used. The experimental protocols were approved (Protocol Number 21.16) and supervised by the Animal Ethics Committee of Vall d'Hebron Institut de Recerca according with the Spanish legislation and the Directives of the European Union. ARRIVE guidelines were followed.

### Mouse Habituation and Permanent Focal Cerebral Ischemia Model

Briefly, C57BL/6 mice were purchased from Janvier Laboratories (Saint Berthevin, France). Mice were housed in a temperature-/humidity-controlled room and maintained on a 12-h light–dark cycle and given water and food *ad libitum*. The habituation protocol for the treadmill was conducted to all mice before ischemia to avoid neophobic behaviors during exercise therapy. Briefly, the week before ischemia and for 3 consecutive days, all mice were placed in a stationary treadmill apparatus 10 min/day (from 3:00 to 6:00 p.m.).

The distal occlusion of the middle cerebral artery (MCAo) was conducted under body temperature and cortical cerebral flood flow (CBF) monitoring as described (25). Animals were anesthetized with isoflurane (Abbot Laboratories, Spain) for a maximum of 30 min via face mask (4% for induction and 1–2% for maintenance in Medicinal Air, 79% N_2_/21% O_2_), and eyes were protected using an ophthalmic ointment (Lipolac™, Angelini Farmaceutica, Barcelona, Spain). A small craniotomy was performed between the eye and ear area to expose the distal part of the MCA after temporal muscle retraction. The MCA was compressed using a 30-G needle and indirectly electrocauterized by heating the compressing needle. CBF was monitored using a laser-doppler flowmetry (Moor Instruments, Devon, UK), and only animals with a reduction in CBF below 80% were included. Buprenorphine (0.05 mg/kg) was administered subcutaneously during surgery, the skin was sutured, and mice were allowed to recovery from anesthesia under body temperature control.

### Pre-clinical Treadmill Rehabilitation

For the study of SVZ neurogenesis, rehabilitation began 48 h after MCAo and consisted of 12 days of treadmill exercise or non-exercise (No-RHB). For treadmill, mice received 30 min of exercise by increasing the speed every 10 min (10, 15, and 20 cm/s) without any aversive stimulus (such as the electric shock), and a plastic barrier was placed between the shock grid and the treadmill line to prevent animals from resting on the top of the grid during the rehabilitation protocol. The No-RHB group was placed at the treadmill apparatus (0 cm/s) for 30 min the same days of treatment, but only free movements were allowed.

For the ANG molecular analysis of the SVZ, a new group of mice were habituated to the treadmill as described above, and 48 h after pMCAO, mice received treadmill rehabilitation or no rehabilitation for 3 consecutive days. The day after the last session, mice were euthanized for brain processing.

### Infarct Volume Assessment

During the euthanasia procedure, brains were removed by intracardiac perfusion with cold saline and under deep anesthesia as described. Brains were cut into 1-mm-thick coronal sections and stained with 2.5% of 2,3,4-triphenyl-2H-tetrazolium chloride (TTC; Sigma, St. Louis, MO, USA) for 10–15 min at room temperature when TTC solution was replaced by cold saline, and images were acquired for infarct quantification by the ImageJ free software as described previously correcting for brain edema (21).

### Angiogenin ELISA

To determine the angiogenin levels in the SVZ, we dissected two middle sections of TTC-stained brains corresponding to the SVZ area along the wall of the lateral ventricles of the ipsilateral and contralateral hemispheres. Tissues were snap frozen in dry ice and stored at −80°C. Brain homogenates were prepared with 150 μl ice-cold lysis buffer (50 mM Tris–HCl, 150 mM NaCl, 5 mM CaCl_2_, 0.05% BRIJ-35, 0.02% NaN_3_, 1% Triton X-100, 1% phenylmethanesulfonyl fluoride, and 0.5% aprotinin), and protein content was collected from the supernatant after centrifugation at 15,000 g for 12 min at 4°C. Total protein was determined by duplicate in each sample by the bicinchoninic acid (BCA) assay (Thermo Fisher Scientific Inc., Waltham, MA, USA). Finally, Mouse Angiogenin SimpleStep ELISA® Kit (ab208349, Abcam, Cambridge, UK) was used following manufacturer's instructions (sample dilution 1/5, and coefficient of variation of replicates <25%). Data are expressed as picograms of angiogenin per microgram of total protein per sample.

### Immunohistochemistry

Mice of the SVZ neurogenesis study received daily intraperitoneal injections of 5-bromo-2′-deoxyuridine (BrdU, 50 mg/kg in saline, B9285, Sigma-Aldrich, St. Louis, MO, USA) beginning 48 h after MCAo until euthanasia to label proliferating cells. For euthanasia, transcardial perfusion with cold paraformaldehyde (4% PFA) was performed under deep anesthesia (isoflurane). Brains were removed and fixed with 4% PFA for 2 h, followed by 30% sucrose for cryoprotection, embedded in optimal cutting temperature (OCT) (Tissue-Tek, Fisher Scientific, Waltham, MA, USA), and frozen at −80°C until use. Slices (12-μm thick) were cut in a cryostat, placed at room temperature for 30 min, washed three times [0.1% phosphate-buffered saline (PBS)-Tween, 0.3% PBS–Triton X-100, and 0.1% PBS–Tween] and further incubated for 1 h with 2 M HCl-PBS followed by 10 min in 0.1 M borate buffer and 5 min in 0.1% PBS–Tween for the detection of nuclear BrdU of dividing cells. Sections were blocked using 0.1% PBS–Tween containing 1% BSA (Sigma-Aldrich, St. Louis, MO, USA) and 5% goat serum (Merck Millipore, Billerica, MA, USA) for 1 h. Then, slices were incubated with the following antibodies, namely, 1:400 rabbit anti-DCX (ab18723, Abcam, Cambridge, UK), 1:100 mouse anti-DCX (sc-271390, Santa Cruz Biotechnology, Santa Cruz, CA, USA), 1:100 rat anti-BrdU (ab6326, Abcam, Cambridge, UK) or 1:100 rabbit anti-angiogenin (NBP2-41185, Novus, Centennial, CO, USA), or 1:100 mouse anti-nestin (556309; BD Biosciences, San Jose, CA, USA), and washed three times with 0.1% PBS–Tween prior to secondary antibody incubation. Alexa fluor 488 goat anti-rabbit IgG, Alexa fluor 488 goat anti-rat IgG, Alexa fluor 647 goat anti-rabbit IgG, or Alexa fluor 633 goat anti-mouse IgG (Invitrogen, Carlsbad, CA, USA) were used as secondary antibodies at 1:500 for 1 h at room temperature and washed with 0.1% PBS–Tween. Finally, sections were mounted in Vectashield™ with 4′,6-diamidino-2-phenylindole (DAPI) (Vector Laboratories, Burlingame, CA, USA) and visualized using an Olympus BX61 (Olympus, Tokyo, Japan) or confocal laser scanning (FV1000, Olympus, Japan) microscopes.

Two brain slices per animal were imaged for the entire dorsolateral SVZ for the analyses. The total area of DCX+ fluorescence and double-positive cells (DCX+/BrdU+) was calculated using ImageJ software by an investigator who was blinded to the treatment group.

### Neural Stem Cells

**Figure 1:**
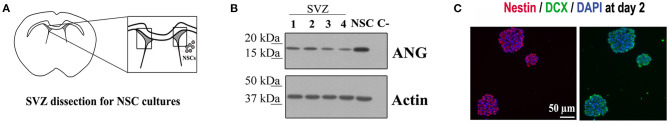
Angiogenin expression in the SVZ niche: **(A)** schematic figure of the SVZ dissection for NSCs isolation. **(B)** Representative image of Western blot proving the presence of angiogenin in SVZ niche of different naïve mice and in primary NSCs in culture derived from the adult SVZ (see online Supplementary Material for the full-length Western blot film); **(C)** Neurosphere immunocytochemistry phenotyping showing expected nestin and DCX markers. ANG, angiogenin; C–, negative control (NSC culture media); DCX, doublecortin; NSCs, neural stem cells; SVZ, subventricular zone.

NSCs were obtained from the SVZ as previously described (24), Figure 1A. Frozen NSCs were thaw at 37°C and cultured in a mixture of 1:1 Dulbecco's modified Eagle's medium (DMEM) and F12 (Gibco, Thermo Fisher, Waltham, MA, USA) supplemented with 0.25% of P/S, 8 μg/ml of heparin (H-3149; Sigma-Aldrich, St. Louis, MO, USA), 0.02 μg/ml of hFGF-B (PHG0024; Thermo Fisher, Waltham, MA, USA), 0.02 μg/ml of hEGF (PHG0314; Thermo Fisher, Waltham, MA, USA), 2% of B27 (12587010; Thermo Fisher, San Jose, CA, USA), and 1% of L-glutamine (25030149; Thermo Fisher, San Jose, CA, USA). After 2 days in culture, 3D-proliferating structures known as neurospheres were observed (Figure 1C).

For phenotyping purposes, growing neurospheres were stained for nestin and doublecortin (DCX) at day 2. Briefly, neurospheres were fixed with 4% PFA for 10 min followed by three washes with 1 × Dulbecco's phosphate-buffered saline (DPBS) and blocking with DPBS–Tween with 1% BSA (Sigma-Aldrich, St. Louis, MO, USA) for 1 h. The primary antibodies mouse anti-nestin (1:200, BD-556309, BD Biosciences, San Jose, CA, USA) and rabbit anti-DCX (1:200, ab18723, Abcam, Cambridge, UK) were incubated overnight. Cells were washed three times, and the secondary antibodies Alexa Fluor 568 goat anti-mouse IgG and Alexa Fluor 488 goat anti-rabbit IgG, respectively, were added for 1 h. Finally, cells were mounted in Fluoroshield™ with DAPI, and pictures were acquired using the Leica DM IRBE (Leica Microsystems, Wetzlar, Germany).

### NSC Counts and Neurosphere Growth

**Figure 2:**
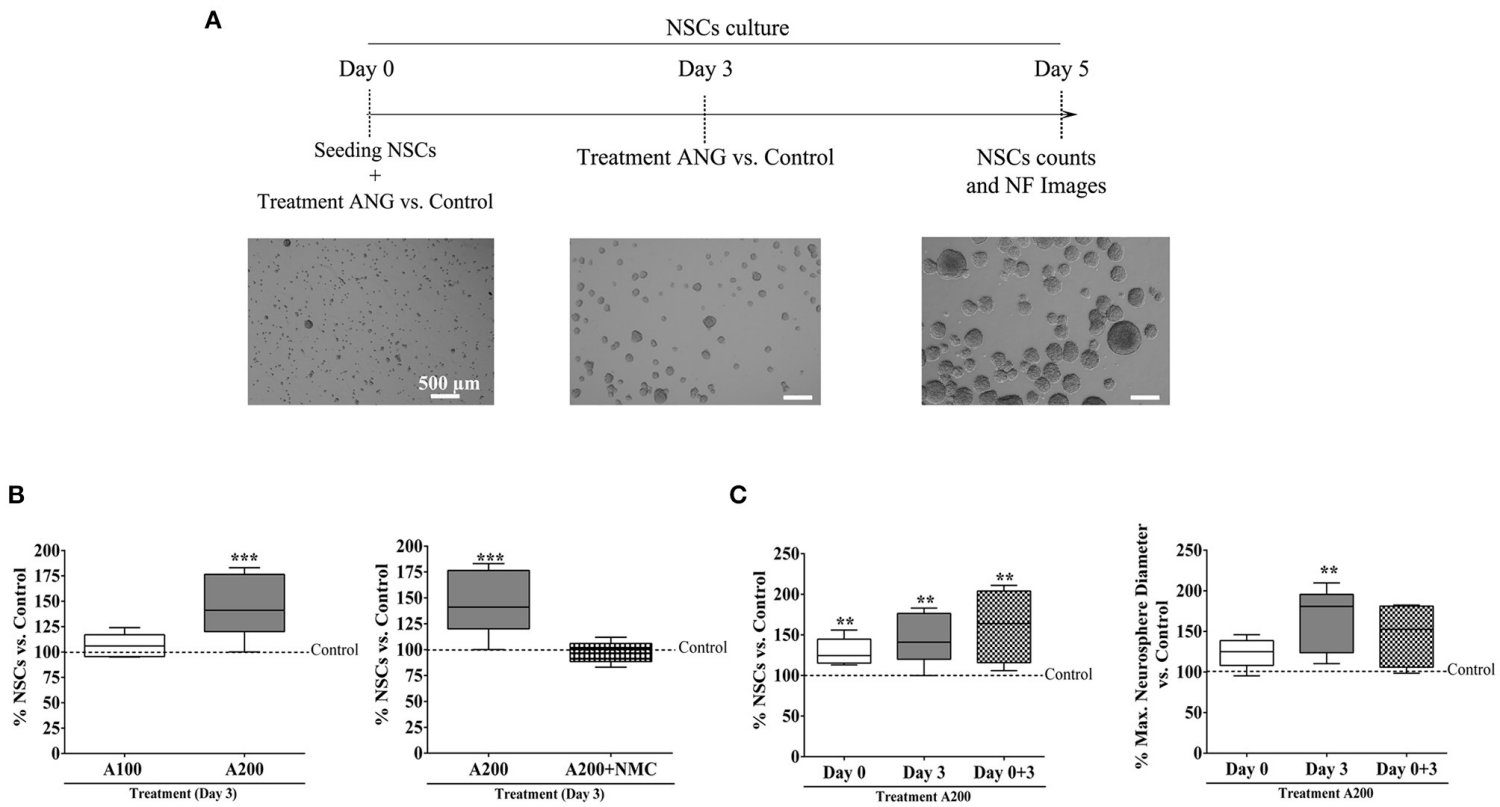
SVZ-derived NSCs responses to angiogenin: **(A)** timeline of the experimental procedure of neurosphere cultures and representative images; **(B,C)** box plots showing quantification of the NSC-forming neurospheres after angiogenin stimulation (100 or 200 ng/ml) at different time points, the inhibition with neomycin, and the largest neurosphere diameters obtained with angiogenin treatment; *n* = 4–9; **p < 0.001 and ***p < 0.001 vs. control. Box plots represent median (IQR) of the percentage vs. control values of each independent experiment. ANG/A, angiogenin; NSCs, neural stem cells; NMC, neomycin; NF, neurospheres.

Cell cultures of NSCs forming neurospheres were assessed using the trypan blue exclusion method and a hemocytometer to quantify NSCs numbers after angiogenin treatment (Figure 2A). Single NSCs were seeded at a density of 30,000 cells/ml and cultured in uncoated 12-well plates to allow neurosphere formation. Cells were treated on day 0, day 3, or both with 100 or 200 ng/ml of angiogenin. We also treated cells with 100 μM neomycin (a selective inhibitor of angiogenin, which blocks its nuclear translocation) on day 3. Three images per well were captured at ×100 magnification on day 5 of treatment using the Olympus IX71 microscope. ImageJ software was used to measure the neurosphere diameter. Afterwards, the neurospheres were collected and centrifuged at 1,500 rpm for 5 min; the pellet was resuspended in 300 μl of NSCs media and pipetted to obtain single NSCs, which were quantified by the trypan blue method with a hemocytometer. To evaluate the effect of each independent experiment, data are expressed as a percentage of the experimental control condition.

### SH-SY5Y Culture and Neurite Outgrowth

**Figure 3:**
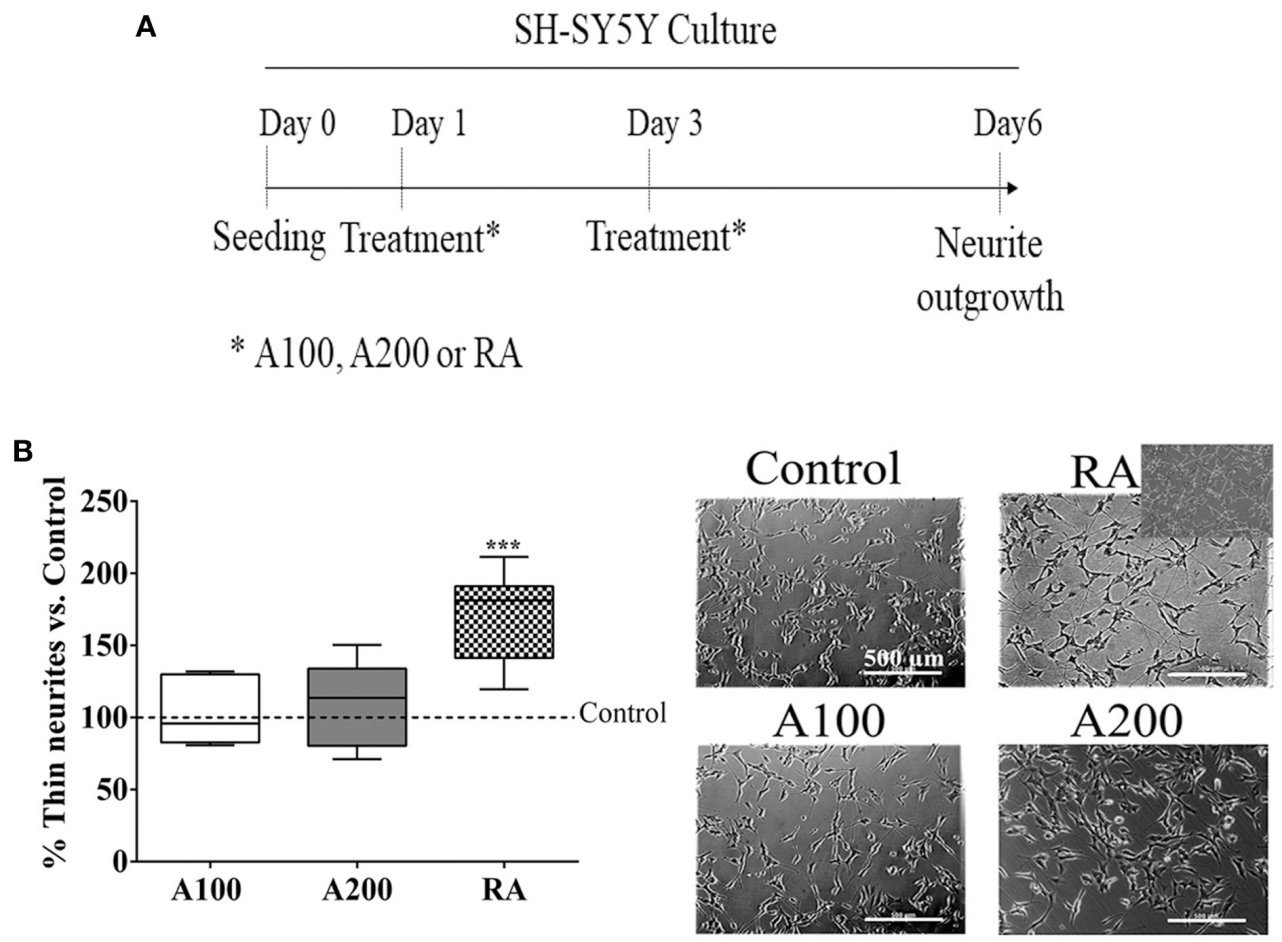
SH-SY5Y neurite differentiation: **(A)** timeline of the experimental procedure. **(B)** Box plots and representative images showing axonal/neurite outgrowth in the presence of Retinoic Acid (RA) as expected, but not with angiogenin stimulation; *n* = 5–6. The insert in RA shows a micrograph representative of the WimNeuron analysis. Box plots represent median (IQR) of the percentage vs. control values of each independent experiment, ***p < 0.001 vs. control.

The human neuroblastoma cell line SH-SY5Y was purchased from ATCC (ATCC® CRL-2266) since they exhibit a neuron-like phenotype with outgrowth neurites in the presence of Retinoic Acid (RA). Cells were maintained in complete medium containing DMEM/F-12 (Gibco, Thermo Fisher, San Jose, CA, USA), 10% fetal bovine serum (FBS), 1% non-essential amino acids (NEA), and 1% penicillin–streptomycin (P/S). We seeded 12,500 SH-SY5Y cells onto collagen type-I-coated 24-well plates with complete media for differentiation. The medium was replaced with differentiation media after 24 h for neurite outgrowth: DMEM/F-12 (Gibco, Thermo Fisher, San Jose, CA, USA), 1% FBS, 1% NEA, and 1% P/S supplemented with ANG (100/200 ng/ml) or 10 μm RA to induce neurite differentiation. The media and treatments were changed after 2 days, and cells were imaged (three fields/well) on day 6 using an Olympus IX71 (×100 magnification), Figure 3A. Finally, WimNeuron automated analysis software (Wimasis Image Analysis®) was used for quantification by measuring the circuitry length and the total thin neurite length. Data are expressed as a percentage of the control condition of each independent experiment.

### Western Blot

Brain dissections from the SVZ of naive C57Bl/6 mice and cell pellets from cultured NSCs were homogenized and lysed with freshly prepared ice-cold lysis buffer containing 50 mM Tris–HCl, 150 mM NaCl, 5 mM CaCl_2_, 0.05% BRIJ-35, 0.02% NaN_3_, 1% Triton X-100, 1% phenylmethanesulfonyl fluoride (PMSF; Sigma-Aldrich, St. Louis, MO, USA), and 0.5% aprotinin (Sigma-Aldrich, St. Louis, MO, USA). Homogenates were centrifuged at 12,000 rpm for 10 min at 4°C and the protein fraction in the supernatants assessed by the bicinchoninic acid assay (Thermo Scientific™, Rockford, IL, USA). A total amount of 10 μg protein was mixed with Laemmli buffer and 5% of 2-mercaptoethanol, heated for 5 min at 95°C, run into 12% polyacrylamide electrophoresis gels, and transferred into polyvinylidene fluoride (PVDF) membranes (Thermo Scientific™, Rockford, IL, USA). Then, membranes were blocked for 1 h with 10% non-fat milk (in PBS, 0.1% Tween 20, Sigma-Aldrich, St. Louis, MO, USA) and incubated overnight at 4°C on a shaker with the following antibodies: anti-angiogenin (1:500, NBP2-41185, Novus, Centennial, CO, USA) or β-actin (1:5,000, A5316, Sigma-Aldrich, St. Louis, MO, USA). The membrane was then washed three times (PBS−0.1% Tween 20) and incubated with corresponding secondary antibodies at 1:2,000 for 1 h at RT with gentle agitation. Finally, membranes were washed three times (PBS−0.1%Tween 20) and briefly incubated with Pierce® ECL Western Blotting Substrate (Thermo Scientific™, Rockford, IL, USA) to visualize the chemiluminescence signal with Fujifilm FPM-100A films. Molecular weight markers were also run for reference values.

### Statistical Analysis

The SPSS 20.0 package was used for all statistical analyses, and GraphPad Software was used for graph representations. The normality of continuous variables was assessed using the Shapiro–Wilk-test (*N* < 30). Normally distributed variables were analyzed using ANOVA (followed by Tukey *post-hoc*), and the Mann–Whitney *U*-test or Kruskal–Wallis-tests were used for non-normally distributed variables. For the analysis of repeated measures, the Wilcoxon test was used in non-normal distributions. Graphs represent means ± SEM or medians (interquartile range, IQR) according to the normal or non-normal distribution of the represented variable, respectively. Extreme values were excluded prior to data analyses of cell cultures using the mean ± 2SD criteria. The results with a *p* < 0.05 were considered statistically significant.

## Results

### Angiogenin Is Expressed in the SVZ Neurogenic Niche and Increases NSCs Yields in Free-Floating Neurosphere

First, we examined for the first time the presence of ANG in this SVZ neurogenic site with the hypothesis that angiogenin could be involved in the regulation of neural precursors, which are known to respond to brain injury or physical exercise. As shown in Figure 1B, SVZ-naive tissues were rich in angiogenin as well as SVZ-derived NSC pools used in the present study for neutrospheres formation, which, in culture, showed typical nestin and DCX markers (Figure 1C).

*In vitro* experiments with primary cell cultures of SVZ-derived mouse NSCs exposed to exogenous ANG were conducted as indicated in Figure 2A. Only the treatment with the highest dose of angiogenin (200 ng/ml) after the neurospheres were formed at day 3 significantly increased NSCs yields (*p* < 0.001), whereas cotreatment with neomycin (a well-known angiogenin activity inhibitor) completely abolished this proliferation (*p* < 0.001); Figure 2B. Indeed, no toxic evidence of neomycin was observed on NSCs cultures (*p* > 0.05; Supplementary Figure 1, Figure 1). We also treated NSCs with the highest angiogenin dose (200 ng/ml) at the beginning of the culture prior to neurosphere formation (day 0), during neurosphere growing (day 3 after seeding), or both, and again, all treatment conditions showed a significant increase in NSCs yields vs. the control (day 0, *p* = 0.002; day 3, *p* = 0.003; and day 0–3, *p* = 0.004) with a visible increase in the neurosphere diameter; Figure 2C. In this regard, the maximum diameter achieved occurred when angiogenin was added to formed neurospheres on day 3 (*p* < 0.01 vs. control) (Figure 2C).

Finally, we investigated whether ANG could also trigger differentiation of a neuron-like cell line (SH-SY5Y cells) by its neurite outgrowth (Figure 3A). However, the capacity of axonal/neurite sprouting was only confirmed in the presence of retinoic acid, as expected (*p* < 0.001), but ANG did not show this mature neuron-like phenotype at any of the tested concentrations (Figure 3B).

### Physical Exercise Increases SVZ Angiogenin After Ischemia in Areas of Active Neurogenesis

**Figure 4:**
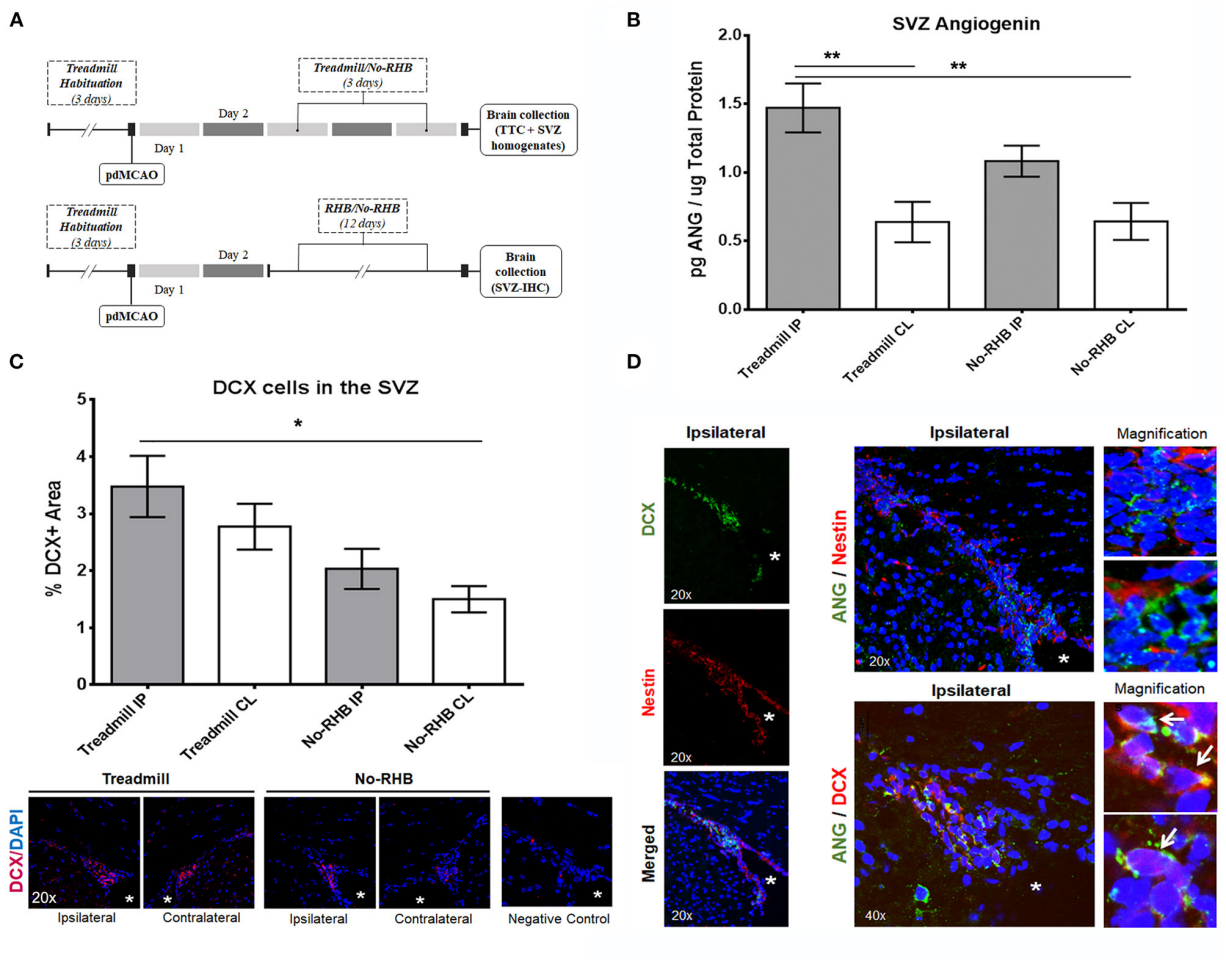
Post-stroke SVZ neurogenesis and angiogenin expression: **(A)** timeline schemes describing the pre-clinical stroke and treadmill rehabilitation protocols. **(B)** Bar graph representing angiogenin after 3 days of treadmill rehabilitation or no rehabilitation in the SVZ area, measured by ELISA (*n* = 6/group); **(C)** bar graph representing DCX+ quantification in the immunohistochemistry study of the SVZ (*n* = 5/group) and representative immunostains of the ipsilateral hemisphere identifying the neuroblasts (DCX+). **(D)** Representative immunostains of the SVZ after stroke and treadmill exercise showing the distribution of nestin+ and DCX+ NSCs in the SVZ (left panel); notice that only neuroblasts (DCX+) had the most angiogenin expression, which is specifically shown in the insert magnifications and in white arrows. Bar graphs represent mean ± SEM, *p < 0.05 and **p < 0.01 as indicated by the horizontal lines. ANG, angiogenin; CL, contralateral; DAPI, 4′,6-diamidino-2-phenylindole; DCX, doublecortin; IP, ipsilateral; RHB, rehabilitation; SVZ, subventricular zone. The white * indicates the position of the lateral ventricle with contiguous dorsolateral SVZ.

Three days after ischemia (Figures 4A,B), brain NAD in the SVZ was altered among the studied areas (*p* = 0.0014), showing the largest amount in the ipsilateral SVZ of the treadmill exercise group (*p* = 0.0032 vs. treadmill contralateral and *p* = 0.0034 vs. No-RHB contralateral SVZs), as seen in Figures 4A,B. This result was not influenced by potential differences in the ischemic lesion, since infarct volumes were similar between groups (treadmill 18.03 ± 3.2 mm^3^ vs. No-RHB 16.23 ± 3.6 mm^3^; *p* = 0.72, see Supplementary Figures 2A,B.

We also evaluated the presence of DCX+ cells in the SVZ, since neurogenesis was expected at later time points (12 days of treadmill exercise, Figures 4A,C). Our results show significant differences among the studied areas (*p* = 0.015) with larger DCX+ signal in the ipsilateral SVZ of treadmill-exercised mice (*p* = 0.08 vs. No-RHB ipsilateral and *p* = 0.013 vs. No-RHB contralateral, as shown in Figure 4C) but not in BrdU+ nuclei (*p* = 0.06, not shown).

The immunohistochemistry study showed that the ANG increase detected by ELISA in the SVZ of treadmill-exercised ischemic mice was localized in DCX+ neuroblasts emerging from the SVZ (Figure 4D), in the vicinity of nestin+ cells, which did not present ANG colocalizations (see magnifications in Figure 4D).

## Discussion

The present investigation focuses on describing angiogenin expression in the adult SVZ and its putative effects on neurogenic responses after stroke. Specifically, we describe that (i) for the first time, angiogenin is expressed in the adult SVZ, (ii) angiogenin increases the NSC yields in SVZ-derived neurosphere cultures, (iii) angiogenin is overexpressed after physical exercise in the SVZ of the ischemic hemisphere during neurogenesis, and (iv) SVZ angiogenin is mainly expressed in DCX+ neuroblasts. Our results position angiogenin in the neurogenic SVZ during stroke recovery, suggesting potential therapeutic interventions in neurorepair beyond the known actions on angiogenesis.

Many people survive stroke but exhibit physical and motor deficits that limit functional independence and quality of life. Current rehabilitation programs are implemented in developed countries to reduce stroke-related disabilities to ultimately compensate for the impaired functions (6–8). Several studies demonstrated that exercise improved functional recovery and activated cerebral-repair-associated processes within a plastic brain (11, 26). These data are supported by pre-clinical rehabilitation models as emerging strategies to investigate underlying mechanisms during the recovery phase of stroke and elucidate the molecular and cellular pathways activated during the rehabilitation therapies received in the clinical setting (27–30). Previous investigations in experimental models have described neurogenesis as a key mechanism regulated after stroke by showing increased neurogenesis in the SVZ and the subgranular zone (SGZ) of the hippocampus or reporting that neuroblasts from the SVZ migrated to infarct boundaries in response to the ischemic injury (31, 32). Additionally, studies in rodents under exercise conditions showed enhanced neurogenesis in the hippocampus (13, 33, 34) related to memory recovery and in the SVZ of ischemic brains (35, 36). According to these data, our post-stroke recovery treadmill moderate exercise also enhanced the DCX pools in the SVZ of the ipsilateral hemispheres. In this pos-stroke SVZ niche, we report for the first time the presence of a unique ribonuclease and potent trophic factor, angiogenin, in migrating neuroblasts in the active SVZ closely associated with other neural stem cell pools. Angiogenin is a ribonuclease protein that promotes cell proliferation and migration, and it is known to be secreted by endothelial cells (17, 20, 37). The actions of angiogenin were first described in tumor angiogenesis (38), but it also acts as a neuroprotectant in neurodegenerative diseases *in vitro* and *in vivo* (39). Furthermore, angiogenin is present during mouse embryogenesis and neuroectodermal differentiation (40), and it is also localized in axonal growth cones and neurites, where its inhibition impacts neural pathfinding (but not in embryonic cell differentiation). Among the multiple implications of angiogenin, the most important function described so far is its regulation of angiogenic-related routes in multiple experimental cell lines (17). Its role in neurogenic mechanisms is still unknown, although a close RNase (RNase A) has been recently described to induce NPC proliferation in embryonic-derived neurogenic cultures (41). In this argument line, other authors recently suggested that angiogenin, together with other proteins, participated in the prevention of neural differentiation of neuroepithelial stem cells (42) or demonstrated increased neurosphere formation in an embryonic carcinoma cell line after the addition of angiogenin in culture (43). Our study confirms the neurogenic actions of angiogenin protein in NSC primary cell cultures from adult SVZ niches for the first time by increasing the number of cell yields in growing neurosphere structures, which was further confirmed by adding neomycin (inhibitor of angiogenin) and suppressing the NSC responses. Importantly, we observe a dose–response effect that should be considered in any future therapeutic study design as well as the fact that the increased number of NSC yields forming neurospheres could respond to both proliferation and cell survival actions of angiogenin treatment. Angiogenin has been recently found to be present in the secretome of EPCs (20), and several authors reported that culturing NSCs from the SVZ with endothelial cells or its secretome maintained the stem-like characteristics and enhanced the proliferation of these cells (44, 45). However, the same studies demonstrated that NSCs in culture with endothelial cells under ischemic conditions migrated and differentiated to a neuroblast-like phenotype, which suggest that this mechanism serves as a repair response for neuronal replacement after injury.

SH-SY5Y cells are a subclone of a human neuroblastoma cell line and exhibit a neuroblast-like phenotype. These cells express a marker of stem cell characteristics (46), namely, nestin, under undifferentiated conditions and differentiate into neurons in the presence of retinoic acid (47), which allows investigations on neuronal differentiation *via* the addition of drugs or molecules. In our study, we could not prove the differentiation of SH-SY5Y cells to a mature neuronal phenotype with neurite outgrowth when treated with angiogenin at the tested doses since neither circuit or neurite length were enhanced in the presence of angiogenin. These results are consistent with a previous report (41) showing that angiogenin was involved in the prevention of neural precursor maturation. Here, we should also acknowledge the existence of biphasic actions of this particular ribonuclease molecule. Initially, stress-induced responses have been extensively described in response to angiogenin actions leading to the cleavage of non-coding transfer RNA (tRNA) anticodons and producing tRNA halves (tiRNA) with cytoprotective actions involving cell survival and antiapoptotic mechanisms (48, 49); however, some recent studies point at existing cytotoxic actions of ANG (50) linked to the absence of the ribonuclease inhibitor protein, RNH1 (51).

In conclusion, the present study identifies angiogenin in the neurogenic SVZ and shows its potential actions on NSCs during neurogenesis. Additionally, in the context of stroke, ANG is overexpressed in the ipsilateral SVZ after pos-stroke exercise coexisting with the migration of SVZ-derived neuroblasts. Overall, our results support further investigations on the molecular mechanisms activated by post-stroke neurorehabilitation and the role of ANG as a therapeutic target, which should be explored *in vivo* in pre-clinical study designs of overexpression/exogenous therapeutic administration of ANG considering the potential interaction with comorbid conditions such as diabetes, age, or hyperglycemia.

## Data Availability Statement

The raw data supporting the conclusions of this article will be made available by the corresponding author, upon reasonable request.

## Ethics Statement

The animal study was reviewed and approved by Ethics Committee of Animal Experimentation of the Vall d'Hebron Research Institute.

## Author Contributions

MG-S, TL, AG, MC, JM, and AR conceptualized and designed the studies. MG-S, AG, TL, CC, EM-G, and AR performed the *in vitro* and *in vivo* experiments/analyzed the data. MG-S and AR contributed to writing of the manuscript. All authors critically reviewed and approved the manuscript.

## Conflict of Interest

The authors declare that the research was conducted in the absence of any commercial or financial relationships that could be construed as a potential conflict of interest.
