# Supplementary material for: Angiogenin in the Neurogenic Subventricular Zone After Stroke
Source: Front Neurol. 2021 Jun 21;12:662235. doi: 10.3389/fneur.2021.662235 (PMC8256153; doi:10.3389/fneur.2021.662235)

SUPPLEMENTARY MATERIAL

Angiogenin in the neurogenic subventricular zone after stroke

Marina Gabriel-Salazar [1,†], Ting Lei [1,†], Alba Grayston[1], Carme Costa[2], Esperanza Medina[1], Manuel Comabella[2], Joan Montaner[1], Anna Rosell[1].

[1]Neurovascular Research Laboratory & Neurology Service, Vall d'Hebron Research Institute, Universitat Autònoma de Barcelona, Barcelona, Spain.

[2] Servei de Neurologia-Neuroimmunologia, Centre d'Esclerosi Múltiple de Catalunya (Cemcat) and Vall d'Hebron Research Institute, Hospital Vall d'Hebron, Universitat Autònoma de Barcelona, Barcelona, Spain.

[†] These autors have contributed equally to this work and share first authorship.

Correspondence: Dr. Anna Rosell, anna.rosell@vhir.org. Vall d'Hebron Research Institute, passeig Vall d'Hebron 119-129, 08035, Barcelona, Spain.

SUPPLEMENTARY METHODS

As explained in the main text some brain samples used for immunofluorescence analysis were obtained from previous study of our group [21]. Those mice were food-restricted for 7 days prior to the habituation procedure. Mice were habituated and trained on the Pasta Matrix Reaching task as described [21] and on the treadmill apparatus. In brief, all mice were offered with four pieces of uncooked pasta per animal (1.6 cm each, Capellini pasta DeCecco) and placed in their housing-cages during 7 consecutive days. To avoid neophobic responses mice were simultaneously habituated to the Methacrylate chamber (20 × 15 × 8.5 cm with two apertures) with small pasta pieces on the floor and the filled pasta matrix structure in front of one of the apertures for three consecutive days (10min/day). After the pasta matrix habituation, limb preference was established by testing the mice for a minimum of 3 days between 9:00 a.m. to 1:00 p.m. Mice were placed in the testing chamber with a matrix full of pasta in the front, and they were encouraged to reach pasta pieces from the aperture for 10min or a maximum 10 attempts. The number of attempts with the right or the left forelimb was recorded and the limb preference was determined by the 70% of limb dominance. After individual laterality preference was established, mice were trained for their preferred forelimb by filling only half of the matrix with pasta (contralateral of the preferred limb). All animals performed at least 10 days of training (5 days/week) or 15 min in the testing chamber. The training was finished the day each mice was able to break a minimum of 9 pieces of pasta consistently for a minimum of 3 consecutive days.

SUPPLEMENTARY FIGURES:

Figure S1: NSCs were treated with 100 μM neomycin (a selective inhibitor of angiogenin) on day 3 to demonstrate no toxic effects of the drug. Data is expressed as absolute cell counts or as percentage of control condition in each independent experiment (n=5; mean±SEM). No statistical differences were observed compared to control media.

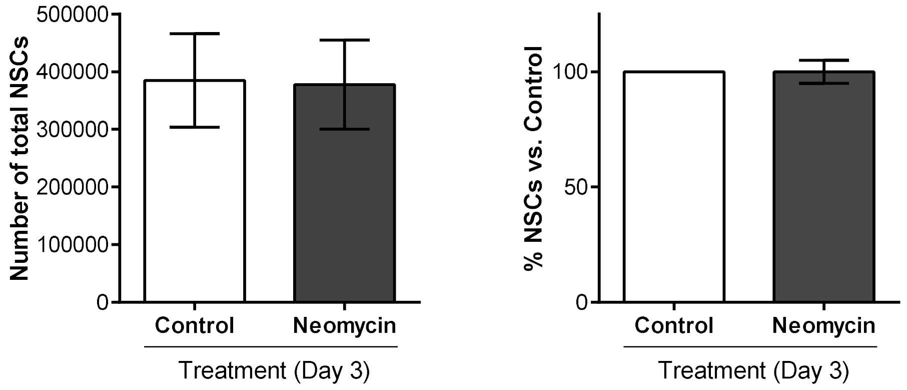

Figure S2: A) Bar graph showing similar infarct volumes after 3 days of physical exercise (treadmill) or no-rehabilitation; n=6/group (mean±SEM) and p=0.7 by t-test. **B)** Representative TTC images showing the infarcted tissue (white cortical areas) in two mice per group.

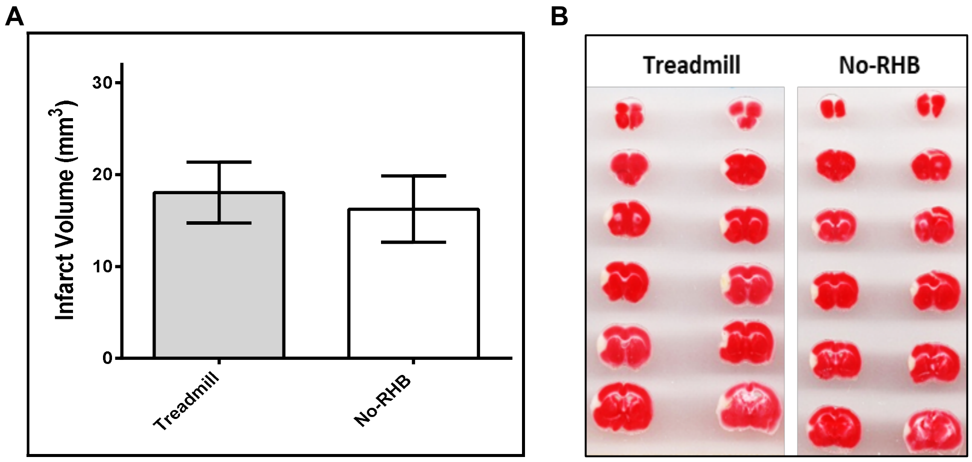

FULL-LENGTH WESTERN BLOT shown in the main text: SVZ tissues from naïve mice and cell lysate from NSC used in the cell cultures. Angiogenin and Actin immunoblotting were conducted and membranes exposed for imaging.

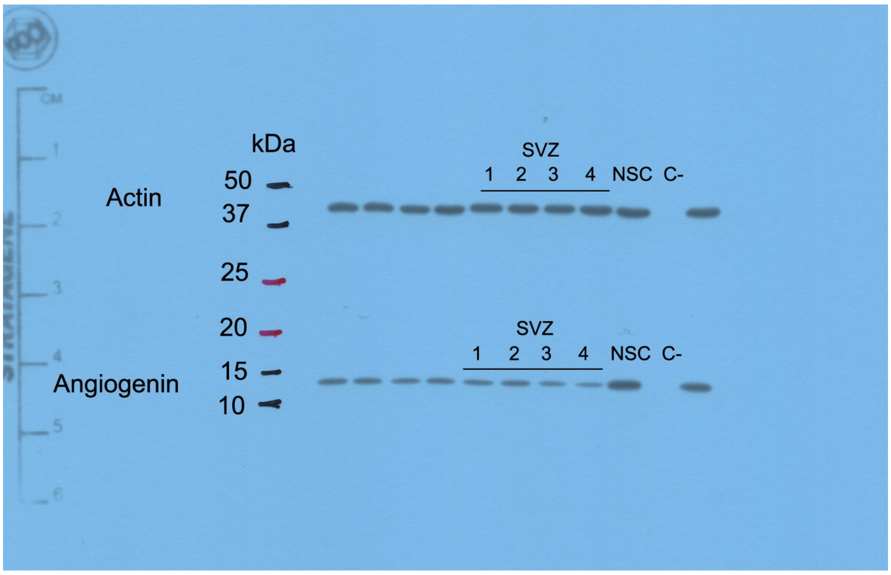

Supplement: Supplementary file 1 [file Data_Sheet_1.pdf]
